# Mathematical Modeling of Complex Biological Systems: From Parts Lists to Understanding Systems Behavior

**Published:** 2008

**Authors:** Hans Peter Fischer

**Keywords:** Systems biology, human biology, complex biological systems, mathematical modeling, computational models, transcriptomics, proteomics, metabolomics

## Abstract

To understand complex biological systems such as cells, tissues, or even the human body, it is not sufficient to identify and characterize the individual molecules in the system. It also is necessary to obtain a thorough understanding of the interaction between molecules and pathways. This is even truer for understanding complex diseases such as cancer, Alzheimer’s disease, or alcoholism. With recent technological advances enabling researchers to monitor complex cellular processes on the molecular level, the focus is shifting toward interpreting the data generated by these so-called “–omics” technologies. Mathematical models allow researchers to investigate how complex regulatory processes are connected and how disruptions of these processes may contribute to the development of disease. In addition, computational models help investigators to systematically analyze systems perturbations, develop hypotheses to guide the design of new experimental tests, and ultimately assess the suitability of specific molecules as novel therapeutic targets. Numerous mathematical methods have been developed to address different categories of biological processes, such as metabolic processes or signaling and regulatory pathways. Today, modeling approaches are essential for biologists, enabling them to analyze complex physiological processes, as well as for the pharmaceutical industry, as a means for supporting drug discovery and development programs.

Over the last decade, DNA-sequencing technologies have advanced tremendously, culminating in the deciphering of the complete human genome in 2001 (Landers et al. 2001; Venter et al. 2001). This achievement is a major milestone in the understanding of human biology, as the human genome provides a catalogue of all human genes and associated molecules that are required for creating a living human being. To date, however, the availability of this “parts” list specifying most human biomolecules, including DNA, proteins, and RNA, has answered only some of the questions concerning the complex phenomena of human biology, leaving many others unanswered. Moreover, the hope that with the knowledge of the human genome sequence researchers would be able to readily develop new therapies for treating human disease as yet has only partially been fulfilled. The availability of a fully sequenced human genome is a prerequisite for elucidating the origins of complex human diseases, such as cancer, obesity, Alzheimer’s disease, or alcoholism but unfortunately is by no means sufficient to provide answers to all of the questions surrounding these diseases.

In the meantime, further technological advances have led to a considerable increase in the understanding of the workings of the human body under normal conditions and in various disease states. For example, transcriptomic^1^ studies are shedding light on which genes are active in a given cell at a given time, proteomic studies are discovering which proteins are present and in what amounts, and analyses of the metabolome have begun to examine which metabolic processes occur under different conditions. Most importantly, however, this work has highlighted the fact that human genes and the proteins they encode do not work in isolation but are connected at various levels in networks and pathways of varying complexity. A deeper understanding of these interactions is pivotal for understanding human diseases and developing appropriate therapeutic approaches. One crucial element in this process is the generation of mathematical models that capture the often-unexpected features of complex biological systems. The development of these models is intimately linked to the generation of experimental data using various high-throughput genomic, transcriptomic, proteomic, and metabolomic experimental strategies.

This article summarizes the challenges associated with the study of complex biological systems, the benefits of systems biology approaches, and the ways in which computational models can help consolidate and interpret the experimental data obtained using these approaches. These principles are exemplified by some concrete examples from current research projects.

## Blueprints of Life: Emergent Properties of a System

The human body consists of approximately 10^14^ individual cells, each of which is itself a complex system comprising thousands of different proteins and other biomolecules. The information specifying the composition and structure of virtually all of these molecules is encoded in the DNA. Although researchers now have information on all genes at hand, they still lack a deeper understanding of many seemingly common biological effects. The reason for this can be exemplified by an analogy with a modern passenger jet, another complex, yet man-made, system. Modern passenger jets consist of thousands of individual components, such as screws, wheels, cables, and other components that perform a specific function in a specific technical context. However, knowledge of those individual components does not reveal functions that arise through interactions with other components, such as those related to takeoff, navigation, communication, or landing. To understand how a plane flies, one must know the interplay of the various components of the plane’s controls and the dynamic regulatory feedback loops that control this interplay. Similarly, the processes that occur in living organisms during growth, metabolism, and regulation of cell functions also are interrelated and require equally tight and coordinated control mechanisms.

The characteristics of a complex system that arise from the interaction of various components are referred to as the emergent properties of the system. Because they are the result of interactions between the different parts, these emergent properties cannot be attributed to any single part of the system. Thus, the ability of a passenger jet to fly is not the consequence of one particular screw (even though this particular screw may be necessary for the plane to function). Similarly, the development of a complex disease (e.g., alcoholism) likely is not caused by a single gene, although a particular gene may be one of the elements necessary for the disease to develop. Such a system is considered irreducible—that is, the system is unlikely to be fully understood by taking it apart and studying each part on its own. To understand irreducible systems and fully appreciate their emergent properties, one must study the systems as a whole.

**Figure 1:**
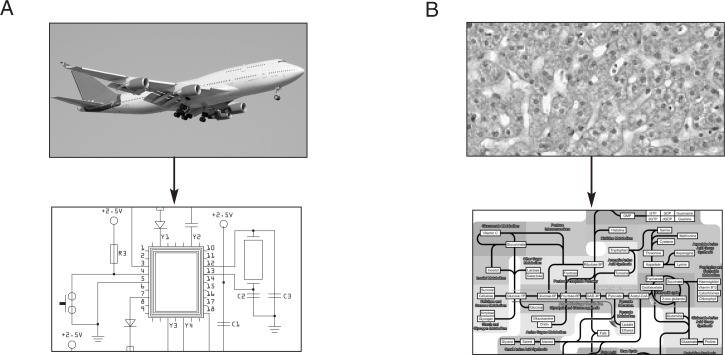
Complex systems and the blueprints used to illustrate the complex interactions that occur between the different components of the systems. **A)** A modern passenger jet (top) is a complex technical system in which the combination of many parts results in complex technical features (emergent properties), such as flying or navigation. Technical blueprints, such as for the microchip used in the jet’s electronic control system (bottom), allow engineers to get an overview on the wiring scheme of the microchip. **B)** Human liver cells (top) are complex biological systems. Pathway maps (bottom) provide a high-level view of the complex networks of biochemical reactions (e.g., for detoxification) within liver cells. These pathway maps help researchers to visualize the interplay of the different molecules and understand the cell’s emergent biological properties. SOURCE: Hepatocytes from http://teaching.anhb.uwa.edu.au/mb140/

The publication of the human genome sequence provided biological scientists with a list of all the individual parts that make up the human body. However, just like having a pile of all the pieces of a passenger jet does not allow a technician to put together a functional plane without having a blueprint of the wiring scheme, this genome sequence is not sufficient to understand the interactions between the genes and their products. Advances over the last few years in transcriptomics, proteomics, and metabolomics that allow investigators to monitor the biological response of cells, however, will allow studies of physiological systems as a whole in order to identify higher-level biological mechanisms encoded in the human genome (Brent 2004) (see figure 1).

## “—Omics” Technologies: The Driving Force Behind Systems Biology

**Figure 2:**
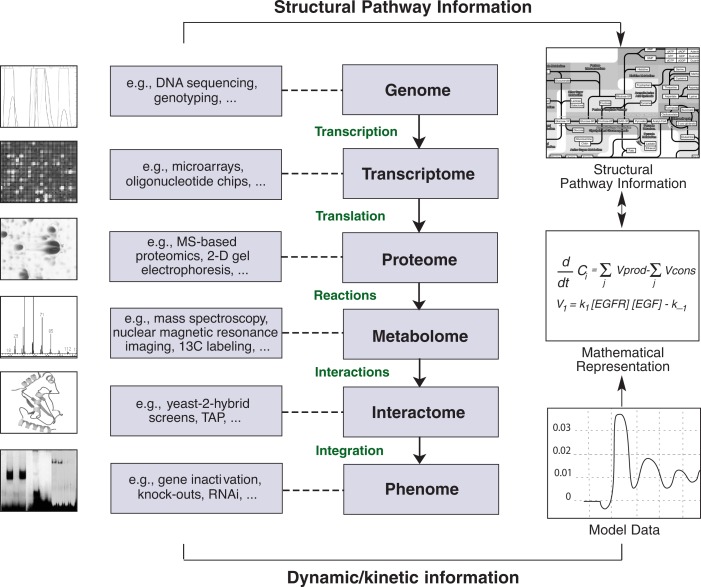
The –omics technologies gather information on numerous levels, including the genome, transcriptome (entirety of all genes that are converted into transcripts [i.e., mRNA molecules]), proteome (entirety of all proteins found in a given cell or tissue), metabolome (entirety of all metabolism products and intermediates in a cell or tissue), interactome (set of molecules, such as biologically active metabolism products, that interact with a given protein), and phenome (entirety of all observable characteristics of an organism) levels. These data are collected using a variety of complementary technologies such as DNA microarrays or mass spectrometry (MS). The experimental data provide the structural and dynamic information that can then be used to generate mathematical formulas representing the observed reactions, leading to the development of comprehensive models and pathway maps. These in silico models allow researchers to evaluate the potential effects of modifications or perturbations in the system and to design further experiments for analyzing additional biological situations (e.g., potential side effects caused by a new drug). SOURCE: Adapted from Fischer, H.P. Towards quantitative biology: Integration of biological information to elucidate disease pathways and drug discovery. *Biotechnology Annual Review* 11:1–68, 2005.

A major reason for the advent of systems biology activities is that only recently analyses at the molecular level of the cell have become technically feasible on a larger scale. With the development of these new, large-scale technologies to identify and quantify molecules on the DNA, mRNA, protein, and metabolite level, researchers for first time are in a position to gather comprehensive data on the molecular state of a given biological system in a systematical manner. These technologies are sometimes collectively referred to as “–omics” technologies (see figure 2). In addition, new techniques to manipulate cells in a directed manner allow researchers to perturb biological systems under controlled conditions. For instance, single genes can be deactivated and the global response of the modified cell can be observed at the protein, transcript, and metabolite level. Together, these experimental techniques allow researchers to obtain a comprehensive picture of the cell’s function as well as of the role of the deactivated gene and its specific function. Such comprehensive and accurate experimental data are critical for developing and testing models of biological processes, and the data produced by –omics technologies are expected to guide the development of new and more complex models.

The new –omics technologies are characterized by three distinct features. First, they allow for analyses on different molecular levels, such as the DNA, RNA, protein, or metabolite level. These different molecular levels sometimes behave asynchronously—that is, although some proteins are highly abundant in a cell, the levels of the corresponding mRNAs from which they are produced may be very low or vice versa. Because asynchronous behavior can indicate the effects of complex regulatory interactions, it is important to examine the role and degree of synchronization of the transcriptome, proteome, and metabolome. For example, metabolites produced in certain biochemical pathways sometimes exert a feedback on key enzymes in the pathway by modifying the expression of the genes that encode these enzymes. The –omics technologies allow researchers to systematically discover such interactions and incorporate them into models aiming to capture the essential regulatory features of a pathway.

Second, –omics technologies are highly parallelized. This means that for a single biological sample many different biological “readouts” can be measured simultaneously. For example, with today’s microarrays researchers can simultaneously measure the expression of virtually all genes of the organism being studied rather than having to perform numerous separate experiments focusing on different genes. This parallelization enables scientists to detect not only the expected but also unforeseen responses of an organism. For example, microarrays in toxicology research allow for a broad screen for unexpected biological side effects caused by new drugs (Ulrich and Friend 2002; Waters and Fostel 2004). The high degree of parallelization also allows researchers to elucidate functional interactions between different genes and proteins and obtain comprehensive images of emergent properties of a cell or organism. This is particularly important for complex cellular processes such as the increase in the number of cells as a result of cell growth and cell division (i.e., proliferation), cell death (i.e., apoptosis), or the response to infection, all of which can involve several hundred different types of molecules.

Third, –omics technologies are highly standardizable and thus amenable to a high degree of automation, allowing researchers to handle and process large numbers of biological samples. The ease of sample processing and experimentation has a huge impact on how experimental studies are performed today. For instance, comparison of larger numbers of replicate experiments allows researchers to validate biological effects with a greater statistical certainty. The ease of use of –omics technologies is particularly relevant for clinical studies, in which large patient populations must be tested to obtain a sound statistical basis for confirming drug efficacy and identifying potential side effects.

The huge amounts of data gathered require tools to automatically process, compare, and interpret the data in a manner that identifies the most relevant pieces of information and which can be used to generate models of, for example, regulatory or metabolic pathways. These tools include computerized data analysis strategies that result in the formulation of mathematical models of the biological systems analyzed.

## Mathematical Models: Tools for Understanding Complex Biological Processes

Biological systems are inherently complex, and many of their emergent properties result from the interplay of numerous molecular components. Moreover, biochemical reactions often obey nonlinear reaction kinetics—that is, an increase in the amount of the starting material of the reaction does not necessarily lead to a proportional increase in the amount of the reaction product. Finally, other complexities, such as cell structure and compartmentalization or random (i.e., stochastic) effects, also often result in unexpected behavior of the entire system. Mathematical models that take these factors into consideration allow researchers to capture the features of complex biological systems and to understand how biological systems respond to external or internal signals and perturbations, such as different growth or development conditions or stress triggered by agents such as alcohol.

Mathematical models have the big advantage of being amenable to computer simulations. Models describing biological systems generally are too complex to be solved analytically (“manually”) and therefore typically are solved numerically—that is, using computers to solve the mathematical equations that help predict the response of a biological system. With the availability of computer-based techniques for solving mathematical equations, the response of a biological system to different conditions can be relatively easily simulated in silico once a mathematical model is available. These computer simulations (so-called “dry experiments”) in many cases require much lower investment and much less time compared with the typically more time-consuming and expensive biological experiments (sometimes referred to as “wet experiments”).

The general approach for creating and using mathematical models in biological sciences is similar to the one followed in other scientific disciplines such as physics and provides the basis for communication between experimental and theoretical scientists. Thus, the theories and mathematical formulas developed by theoretical biologists on the basis of existing experimental data can be tested by experimentalists and used to predict the behavior of biological systems under as-yet-unexplored conditions. Any discrepancies between the predicted and measured results then need to be resolved, either by extending the theoretical framework (i.e., for instance by adding new equations to take into account other apparently important molecules that have not been considered in previous versions of the model) or by refining the experimental setup or data interpretation.

**Figure 3:**
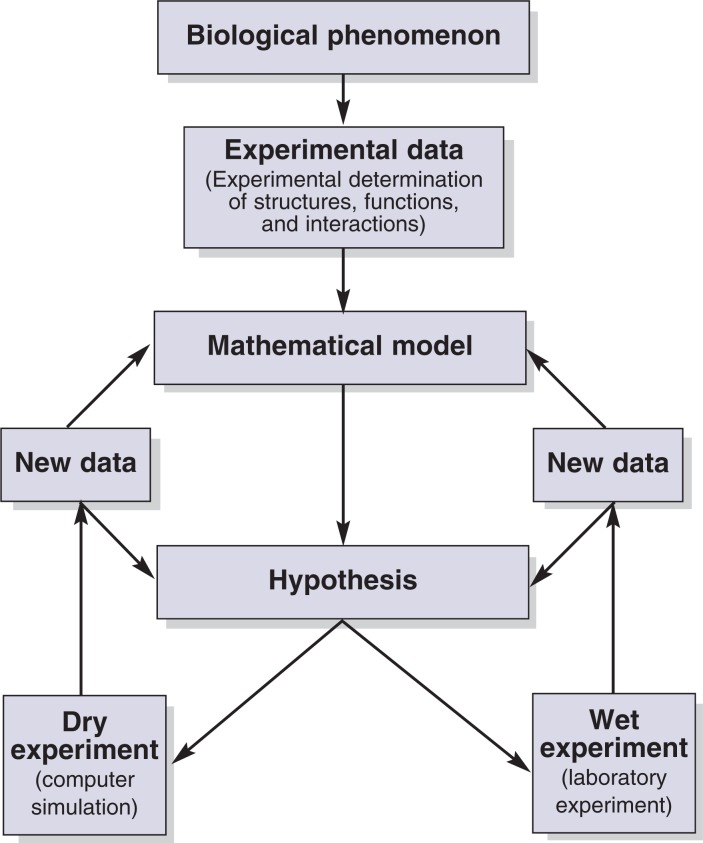
Schematic representation of the process of knowledge generation in systems biology. Experimental data on a given biological phenomenon serve to derive a mathematical model that leads to hypotheses regarding the effects of perturbation of the system. These hypotheses are tested in “dry” and “wet” experiments, leading to the generation of new data that may result in confirmation or modification of the hypothesis and the underlying mathematical models.

Mathematical models for biological systems and the associated computer simulations offer numerous benefits. First, discrepancies between systems behaviors predicted by a mathematical model and actual behaviors measured in experiments can point to components that still are missing from the mathematical model, thereby assisting in developing a more comprehensive picture of a biological process. And even if it is not clear which components are missing from the system under investigation, the results obtained with the mathematical model may help to guide the design of additional experiments to clarify the issue (see figure 3).

Second, mathematical models provide a systematic approach for investigating systems perturbations—for example, those induced by drug administration, genetic alterations, developmental signals, or other factors. To this end, scientists can modify the values of the model parameters (e.g., by introducing modified enzyme activities associated with alcohol administration) and re-run the computer simulations. This approach is relatively straightforward once a reasonable base model is available.

Third, mathematical simulations are not as limited by experimental constraints as wet experiments. Computer simulations can quickly investigate different experimental conditions for the biological system of interest, and only the most relevant cases can be assessed afterwards in the laboratory. This allows researchers to investigate novel scenarios and to develop hypotheses to guide the design of new and promising experiments. This approach is particularly helpful if the wet experiments are difficult and expensive to perform. The combination of dry and wet approaches is at the heart of systems biology and already is being used widely in the metabolic engineering industry, which uses live cells (e.g., bacteria or cultured eukaryotic cells) to produce complex chemicals (e.g., precursors of drugs, vitamins, or amino acids) based on fermentation processes. In these cases, mathematical models are used to suggest directed genetic modifications that may improve the productivity of the microorganisms.

Fourth, mathematical models can be very helpful for systematically determining the relevance of a specific molecule or pathway for the overall behavior of the system. Not all components of a reaction or pathway are equally important, and many biological processes are controlled by relatively small subsystems. Comparison of computer simulations and actual experimental data may help the researcher to readily identify such simpler subsystems that are sufficient to understand the features of the much more difficult-to-treat full biological system.

## Mathematical Equations for Modeling Biological Systems Behaviors

For choosing the optimal modeling approach it is essential to understand the nature of the biological process of interest because different mathematical frameworks have been developed for modeling the behavior of different types of biological systems. For example, most cellular phenomena are governed by dynamic processes so that the cell can adapt to environmental changes or control inherently dynamic cellular functions, such as periodic cell division. For describing such time-dependent phenomena, it is imperative to choose mathematical equations that can capture these dynamic effects. For other biological processes, however, it often is not necessary to describe all the details of the underlying dynamics because some molecules’ concentrations do not change over time (i.e., are quasi-stationary). For most types of applications, appropriate modeling methods have been developed. Two examples are described below—modeling of metabolic processes and modeling of signaling and regulatory pathways.

### Modeling Metabolic Processes

Metabolic processes are essential for all living organisms and provide the cell’s energy, deliver building blocks for the synthesis of larger molecules, or degrade toxic substances. Although biological research has focused on metabolism for several decades, many metabolic processes still are not fully understood, and, in particular, the regulatory mechanisms controlling even well-investigated metabolic pathways often are unknown.

A key parameter in any metabolic study is the metabolic flux—that is, the conversion rate of metabolites along a metabolic pathway. For many research and industrial applications, it is crucial to predict the metabolic flux patterns that indicate which biochemical routes are utilized (e.g., to metabolize nutrients). Modeling techniques are widely used in the field of metabolic engineering to identify any steps in the production of a desired molecule by cultured cells or bacteria that limit the overall rate with which the process occurs (i.e., so-called metabolic bottlenecks). The results of these analyses can guide researchers on how to genetically modify the cells or bacteria to optimize the yield of the desired end product (e.g., see Wendisch et al. 2006). In this setting, changes in metabolic fluxes over time are not a major concern because the fermenters used in the biotech industry typically operate in a steady-state, continuous flow situation and it therefore is sufficient for these applications to consider the fluxes in the cell to be quasi-stationary.

A well-developed set of mathematical methodologies is available for a systematic analysis of such quasi-stationary metabolic phenomena. As an example, consider the process during which sugar is converted to amino acids. In this process, an enzyme called hexokinase adds a phosphate group (i.e., phosphorylates) to the sugar glucose, yielding a compound called glucose-6-phosphate. This reaction must be balanced in terms of atoms and electrical charges. In a chemical notation, the balanced reaction is written as C_6_H_12_O_6_ + ATP^3^ ⇨ C_6_H_11_O_6_PO_3_^2−^ + ADP^2−^ + H^+^. This indicates that both sides of the equation are in a stoichiometric balance—that is, they contain the same number of carbon, hydrogen, or phosphorus atoms. When investigating more complex metabolic networks, each individual chemical reaction contributes a stoichiometric balance constraint that can be formulated as a mathematical equation. However, the individual stoichiometric constraints are not independent from each other. For example, reaction 1 produces a certain number of carbon atoms that then feed into reaction 2, and so on. Consequently, all mathematical equations representing the stoichiometric constraints must be solved simultaneously, and indeed there are many well-developed techniques to solve such sets of equations (for reviews, see Hornberg et al. 2007; Joyce and Palsson 2007). Interestingly, because stoichiometric constraints allow only for relatively few solutions, flux patterns in quasi-stationary networks are relatively limited, which facilitates understanding metabolic processes.

The validity of such mathematical models of metabolic networks can be tested through experiments using substances that are either radioactively labeled or otherwise detectable. For instance, a cell’s carbon flux patterns may be reconstructed by growing the cells in a medium that contains labeled carbon sources (e.g., the so-called ^13^C-method) (Wiechert 2002) that will be taken up and metabolized by the cells. As the labeled substances enter into a metabolic network, their flux across several metabolites can be traced with nuclear magnetic resonance or mass spectrometry. By tracing the labeled atoms across a number of key metabolites, scientists can measure the cellular flux distributions, which can help validate or disprove metabolic network models.

### Modeling Signaling and Regulatory Pathways

Signaling pathways serve as the cell’s central control machinery, which tightly regulates the cell’s response to external and internal stimuli. These pathways involve the transmission of external and internal signals through the cell’s membrane and interior into the cell’s nucleus, where they activate or deactivate specific sets of genes. All of these processes are inherently dynamic events that require different mathematical modeling strategies than the quasi-stationary (metabolic) processes discussed above.

Many signaling pathways are triggered by the binding of extracellular biomolecules (e.g., hormones or growth factors) to a docking molecule (i.e., receptor) embedded in the membrane surrounding the cell. Receptors are proteins, often spanning the membrane, which expose one part of their structure to the exterior environment (i.e., the extracellular space) and one part to the cell’s interior (i.e., the cytoplasm). If a signaling molecule binds to the extracellular region of the receptor, the receptor’s three-dimensional structure may change—a process that can trigger cascades of biochemical reactions within the cytoplasm. These cascades often involve specialized signaling molecules such as enzymes known as kinases, which transfer phosphate groups from one molecule (the donor) to a specific target molecule (the substrate) and which are used extensively to transmit and integrate signals to control complex cellular processes. In many cases, kinases act on and modify the activity of specific proteins. The addition of the phosphate group changes the substrate protein’s biochemical behavior so that it, in turn, can modify additional signaling molecules in the signaling cascade. Ultimately, this chain reaction results in the activation of proteins called transcription factors that bind in the cell nucleus to DNA, triggering expression of distinct sets of target genes. It is this gene activation that alters the cell’s behavior and represents the cell’s response to the initial stimulus.

Obviously, signal transduction is an inherently dynamic phenomenon, as the cell has to be able to flexibly respond to changes within the organism and in the environment. To model the dynamics of signaling cascades such as those described above, researchers primarily rely on so-called differential equations (technically known as time-dependent ordinary or partial differential equations). Such differential equations are used to model the dynamic behavior of, for example, the changes in the concentration of signaling molecules over time as well as the signaling molecules’ distribution across different cellular compartments. Modeling of even relatively simple signaling networks has revealed that signal transmission through the cell often shows unexpected behaviors, such as periodic activation patterns or enhancement (i.e., amplification) of the initial signals (Hoffmann et al. 2002; Swameye et al. 2003). A specific example of how regulatory processes are being modeled is described in the accompanying sidebar, p. 56.

### Identifying Model Parameters

Any equation in a mathematical model will contain one or more parameters that describe certain biophysical characteristics of the molecules involved in the reaction or pathway being studied. For example, when modeling the dynamics of a network of biochemical reactions, the mathematical equations must incorporate parameters that reflect the kinetic properties of the involved enzymes—for example, the number of reactions the enzyme can perform during a given period of time (i.e., the rate constant). Researchers must know these kinetic parameters before they can set up well-defined systems of differential equations representing appropriate models of biological processes.

In principle, the kinetic parameters for all the relevant enzymes can directly be experimentally determined. In practice, however, many kinetic parameters, even for otherwise well-investigated enzymes, still are unknown, primarily because the relevant experimental data are lacking (i.e., the actual direct measurements of enzyme activity have not yet been performed). But even if the kinetic parameters have been measured, these data often are based on experiments performed in a test tube with purified enzymes (i.e., in vitro), and it is unlikely that the enzymes behave similarly under the conditions found in a living cell (i.e., in vivo). To overcome this limitation, one can use dynamic measurements of the overall system. Computational procedures are available to estimate the appropriate model parameters by testing different parameter sets until they fit the available experimental data. However, this process is critically dependent on the quality of the experimental data, and unreliable experimental data will lead to unreliable predictions of kinetic parameters and therefore to models of very limited value (Schilling et al. 2005). The lack of sufficiently large, high-quality experimental datasets still is a major hurdle in developing models even for relatively simple signaling networks.

It must be pointed out that some biological phenomena cannot be described by using models that are just based on continuous concentration fields (described, for example, by partial differential equations), ignoring the existence and relevance of individual molecules. For instance, on a molecular scale random molecular movements can have a major impact on cellular processes. The molecules in a cell are closely packed, and thermally induced random movements of these molecules as well as interactions between them can strongly affect the transmission of signals. To account for such effects, a random (i.e., stochastic) component must be integrated into the mathematical equations of the model. Particularly for rare signaling molecules, of which only a few copies may be present in the cell, the effects of such a stochastic component are significant and must not be neglected.

Another issue that complicates modeling of biological systems is that the different components of a pathway often act over different time and distance scales. For example, metabolic reactions can happen within seconds or minutes, whereas genetic regulatory processes that are induced by these metabolic reactions may occur over several hours or even days. Similarly, some regulatory reactions occur over small distances (e.g., across the width of the membrane surrounding a cell), whereas others involve greater distances (e.g., the entire cytoplasm of the cell, or between tissues where blood circulation may transport signaling molecules such as hormones and activate specific cell types in remote tissues). To couple relevant effects occurring on very different length and time scales, researchers use so-called multiscale models as a compromise to avoid making the model overly complex.

**Example for the Mathematical Modeling of a Signaling Pathway**

One important signaling factor is nuclear factor kappa B (NFκB), which regulates numerous processes relevant for inflammatory reactions, immune responses, cell proliferation, and survival of the cell. NFκB is a transcription factor—that is, it activates specific genes required for these processes by binding to the DNA in front (i.e., upstream) of these so-called target genes.

Because of its important functions in the cell, the levels and actions of NFκB must be tightly regulated. NFκB normally is found in the fluid filling the cell (i.e., the cytoplasm) and must move to the nucleus to exert its effects on its target genes. To prevent inappropriate movement of NFκB to the nucleus, the molecule normally is linked to a phosphate group (i.e., is phosphorylated) and interacts with proteins called inhibitory molecules of NFκB (IκBs). When the NFκB pathway is activated by another signaling molecule, IκBs are degraded, allowing NFκB to move to the nucleus and activate its target genes. One of these target genes codes for one of the inhibitory molecules, IκBα. Thus, NFκB activation leads to production of new IκBα, which can then bind to NFκB and shut off the NFκB pathway. This is known as a negative feedback loop. Continuous cycles of IκBα degradation and synthesis give rise to regular changes (i.e., oscillations) in NFκB activity that can be observed experimentally. Mathematical models have been used to investigate these oscillations in more detail, and comparisons between “dry” and “wet” experimental results generally have shown good agreement. The mathematical equations for modeling NFκB activation also have been used to identify potential contributions of other molecules that are thought to modulate the NFκB/IκB-mediated regulatory process. For example, modeling approaches using experimental data derived from mice in which specific IκB genes had been inactivated (i.e., knocked out) demonstrated that certain IκBs had a damping effect on cyclic NFκB/IκBα oscillations (i.e., that the changes in NFκB/IκB activity became smaller over time) (Hoffmann et al. 2002).

**Figure:**
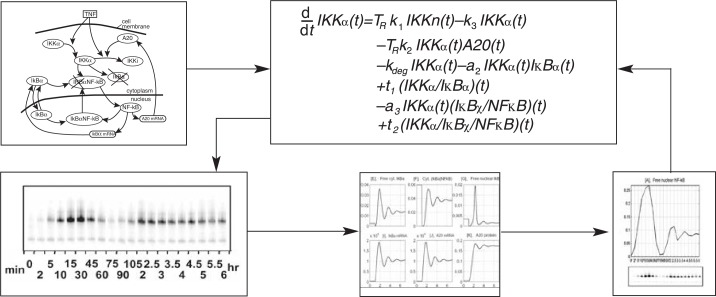
As shown in the upper left panel, NFκB is part of a complex regulatory network that is, in part, controlled by the inhibitory factor IκBα. The cellular concentrations of the relevant molecular players over time can be expressed by formulas such as the one shown in the upper right panel. Experimental analyses of NFκB/IκBα activity over time found that the changes in NFκB/IκB activity became smaller over time (bottom left panel), in agreement with the predictions of the mathematical model. This temporal behavior of the pathway also can be expressed graphically (bottom middle panel), and detailed analysis of these experimental data and resulting graphs (bottom right panel) led to further refinement of the mathematical model. SOURCES: Hoffman et al. 2002, Lipniacki et al. 2004.

Finally, it is important to recognize that any model can be only as good as the assumptions upon which it is based. By definition, any model is an abstraction and simplification of reality. All currently available, tractable mathematical models of biological systems neglect the majority of the molecular players in an organism. Moreover, all models make assumptions about relevant time and length scales. For example, the discussion in this article focuses on modeling approaches for cellular pathways that neglect molecular details (e.g., the exact location of the molecules in the cell, or the precise three-dimensional structure of the proteins). It is essential to keep these limitations in mind when discussing the value and validity of mathematical models or when developing a specific model to capture the characteristics of a given biological process.

## Outlook: Biomedical Applications of Systems Biology and Modeling

Systems biology approaches have great potential for enhancing the drug discovery and development process. Currently, the productivity of the pharmaceutical industry is lagging behind its investments in research and development. The output of new chemical entities (“NCEs,” rather than “me-too” drugs that represent only minor modifications of already existing drugs), which is considered an indicator of the innovation potential of pharmaceutical research, is only slowly growing (Lindsay 2003). The whole process of developing, testing, and obtaining approval for a new drug now costs an average of approximately $900 million for each drug that makes it to the market (Service 2004) and the process can take more than a decade. As a result, companies look for more cost-effective and less time-consuming alternatives to the traditional drug discovery and development process.

Systems biology is expected to play an increasingly important role in the establishment of innovative drug discovery strategies. Today, the industrialized drug discovery process relies, to a large degree, on highly automated processes for screening the biological activity of large chemical libraries in order to identify new drug candidates. These screening assays enable a systematic assessment of candidate compounds by testing their activity on therapeutic targets—that is, biomolecules that are causally involved in disease outbreak or disease progression (Fischer and Heyse 2005).

Systematic compound screening provides the starting point for further chemical development to optimize the drug-like features of the lead compound. Computational models of disease-relevant pathways that allow for dry experiments to support the assessment of a candidate molecule’s safety and efficacy most likely will play an increasingly important role in this process, and the pharmaceutical industry is greatly interested in establishing new predictive, computer-supported methods to systematically identify the most promising drug candidates. In this context, a number of pharmaceutical companies recently have initiated dedicated systems biology programs to support their in-house drug discovery and development programs (Mack 2004).

At the same time, an increasing number of publicly and privately funded initiatives and consortia aim at establishing systems biology in different domains of biological research to elucidate, for example, the functions of specific cell types relevant for medical applications or to develop comprehensive systems biology applications for metabolic engineering. In most cases, multiple research centers or companies are integrated in a decentralized research network to which investigators from numerous disciplines (e.g., biology, genetics, biochemistry, physics, mathematics, computer sciences, statistics, or engineering) contribute (see table, p. 58). One of the pioneering systems biology programs is the publicly funded German HepatoSys initiative, in which more than 40 German research centers cooperate in a joint program investigating the systems biology of the liver cell using numerous complementary approaches. The liver is an organ of great medical interest with relevance to many disease areas, including alcohol-related diseases. The different groups participating in HepatoSys apply a broad spectrum of technologies, ranging from classical cell biological methods to imaging, transcriptomics, proteomics, and metabolomics; in addition, several groups conduct dry experiments by applying pathway modeling to liver cell pathways. Within the overall consortium, different technology platforms are organized into subgroups, or networks, which focus on specific medically relevant themes, such as liver regeneration or detoxification processes. In a first step, the researchers seek to reconstruct liver-specific pathways using wet and dry methods. Later, liver metabolism and liver-specific signaling and their roles in liver regeneration and drug metabolism will be investigated.

For HepatoSys and other systems biology consortia and research centers, it is essential that the different research groups are connected via a central, dedicated data transfer and exchange infrastructure to facilitate the close collaboration among groups and disciplines. Such an infrastructure must include a specialized data management system that allows the participating groups to deposit their wet and dry data in a central location, which is accessible to the experimentalists as well as to the modelers. Although the focus of publicly funded systems biology consortia primarily is on basic research, the results produced will likely also be critical for applications in the healthcare and biotechnology sectors.

In summary, the various systems biology approaches developed in recent years provide an extraordinary amount of new information on many functions of human and other organisms relevant to research and biotechnology applications. Only with the help of computerized modeling efforts, however, can researchers make sense of all the different bits and pieces of information that are accumulating at an ever-increasing pace. Just as an engineer can turn a heap of metal pieces, wires, screws, and bolts into a jet plane only if he has a comprehensive blueprint, biomedical scientists can understand the workings of living organisms only if they have comprehensive models that enable them to connect the often disparate pieces of information derived from experimental approaches. The Greek philosopher Aristotle said more than 2,000 years ago, “The whole is more than the sum of its parts,” and today, systems biology research and modeling efforts are helping to obtain a more comprehensive understanding of life.

## Figures and Tables

**Table:** Overview of Selected Systems Biology Consortia and Research Centers*

**Consortium/Center**	**Country**	**Goal/Background**	**Participants**	**Link**
HepatoSys	Germany	Systems biology of the liver cell	German research centers	www.systembiologie.de
SysMap	Germany	Metabolism of microbial amino acid producers	German industry and academic institutions	
Kluyver Centre	Netherlands	Improvement of microorganisms for use in industrial fermentation processes	Dutch academic institutions and industry partners	www.kluyvercentre.nl
BaSysBio	Nine European countries	Global transcriptional regulation in bacteria	15 European research organizations	www.basysbio.eu
SysMo	Six European countries	Dynamic molecular processes going on in single-cell microorganisms	More than 50 working groups	www.sysmo.net
Manchester Interdisciplinary Biocentre	United Kingdom	Cross-disciplinary approaches to diseases such as cancer, malaria, Alzheimer’s, and cystic fibrosis	Multidisciplinary research groups	www.mib.ac.uk
Institute for Systems Biology	USA	Study of biological systems to increase understanding of the immune system and other biological systems	Multidisciplinary research groups	www.systemsbiology.org
MIT Computational and Systems Biology Initiative	USA	Systematic analysis of complex biological phenomena	More than 10 academic units across the Massachusetts Institute of Technology (MIT)	www.csbi.mit.edu
Kitano Symbiotic Systems Project	Japan	Understanding of system-level principles of biological systems		www.symbio.jst.go.jp

* Links to additional groups involved in systems biology research can be found at http://www.systembiologie.de/de/links_researchgroups_international.html

NOTE: Most consortia are publicly funded on a national or transnational level; some also are co-funded by industry partners.

## Footnotes

1 For a definition of this and other technical terms, see the glossary, p. 84.
